# Distinct trajectories of leisure time physical activity and predictors of trajectory class membership: a 22 year cohort study

**DOI:** 10.1186/1479-5868-5-57

**Published:** 2008-11-07

**Authors:** Tracie A Barnett, Lise Gauvin, Cora L Craig, Peter T Katzmarzyk

**Affiliations:** 1 Centre de recherche du Centre Hospitalier Universitaire Sainte-Justine, 3175, Côte-Sainte-Catherine Montréal (Québec), H3T 1C5, Canada; 2 Département de médecine sociale et préventive, Université de Montréal, Canada; 3 CRCHUM-Centre de recherche du Centre Hospitalier de l'Université de Montréal, Centre de Recherche Léa-Roback sur les Inégalités Sociales de Santé de Montréal, Canada; 4 Canadian Fitness and Lifestyle Research Institute, School of Public Health, University of Sydney, Australia; 5 Pennington Biomedical Research Center, Louisiana State University System, Baton Rouge, LA, USA

## Abstract

**Background:**

Prospective studies linking social factors to long term patterns of physical activity are lacking. In this 22 year longitudinal study, we seek to identify long term patterns of involvement in leisure time physical activity (LTPA), and explore socioeconomic and demographic predictors of distinct LTPA trajectories.

**Methods:**

Among 2102 individuals aged 18–60 years in 1981 who participated in the 1981 Canada Fitness Survey/1988 Campbell's Survey of Well-Being, 1186 (56.4%) completed questionnaires for the 2002/04 follow-up. Complete data on LTPA at all 3 surveys were available for 884 participants. Latent class growth analysis was used to identify major classes of LTPA trajectories; predictors of class membership were identified using polytomous logistic regression.

**Results:**

Four latent classes were identified: *inactive*, *increasers*, *active*, and *decreasers *(53%, 26%, 12%, and 9% of participants, respectively). Women, older participants, those with lower household income, and with lower educational attainment, were significantly less likely to follow *active *(Vs. *inactive*) trajectories of LTPA. Disadvantaged groups with respect to education and income were also significantly more likely to follow *decreasing *(Vs. *active*) trajectories.

**Conclusion:**

There is a need for continued efforts to increase overall population levels of LTPA, particularly among socially disadvantaged groups with respect to income and education, who are most likely to experience unfavorable trajectories of LTPA.

## Background

The evidence is unequivocal that physical activity is strongly and causally associated with health in adults.[1] It has also been convincingly established that social inequalities exist in the population distribution of physical inactivity, with women, older persons, and socio-economically disadvantaged persons pursuing more sedentary lifestyles.[2,3] Though concurrent associations have been widely and consistently reported, prospective studies linking socioeconomic and demographic factors to future levels of physical activity are fewer and their findings appear contradictory. [4-7] This may be due in part to methodological differences, including the measurement and treatment of physical activity indicators in the analysis (e.g. single assessments, averaging repeat assessments, relative change over time), the length of follow-up, and the characteristics of the participants. Either by design or due to statistical limitations, physical activity is not treated typically as a behavioural process that evolves over time. In this study, we sought to identify distinct long term patterns of leisure time physical activity (LTPA), in a large, diverse cohort of adults who provided 3 measures of LTPA over a 22 year period. We then examined socioeconomic and demographic predictors of following specific trajectories of LTPA involvement. These objectives were undertaken using data from the Physical Activity Longitudinal Study (PALS).[8]

## Methods

### Participants

The cohort for this analysis is comprised of individuals who participated in each of three surveys: the 1981 *Canada Fitness Survey *(CFS),[9] the 1988 follow-up *Campbell's Survey of Well-Being in Canada *(CSWB),[10] and most recently, the 2002–04 *Physical Activity Longitudinal *Study.[8] Methods for each survey have been published previously and are briefly summarized herein. The 1981 CFS was designed to describe fitness and physical activity levels of Canadians, and included approximately 23 000 individuals aged seven years and older selected from a geographically based, random sample of households. The 1988 CSWB sampling frame comprised 20 percent of CFS participants who were randomly selected from 61 of the original 80 geographical areas; areas were selected to ensure proportionate regional representation. Participants in the CFS/CSWB were eligible members of the PALS with the exception of 14 individuals who had left the country and 55 individuals who could not provide information without assistance due to language barriers. All individuals aged 18–60 years in 1981 who completed questionnaires both in 1981 and in 1988 were potentially eligible to participate in the current study (n = 2389). Although the cohort established in 1988 was extended to include new family members who subsequently became eligible to be part of the PALS,[8] new members were not eligible for the current study. Initial tracing procedures identified 265 individuals who were reported to be deceased and 22 who were unable to participate for health reasons. Of the remaining 2102 individuals, 510 could not be traced and/or contacted, 406 were traced but refused, and 1186 (56.4 percent) completed questionnaires. Of these, 302 were excluded due to missing data on LTPA for one or more years. Thus the final cohort retained for this analysis included 884 adults aged 18–60 years in 1981, clustered into 644 families (including 422 single-member families, 206 two-member families, 14 three-member families, and two four-member families). Baseline characteristics of participants retained for this analysis and of eligible non-participants (n = 1574) are shown in Table 1.

**Table 1:** Baseline characteristics of PALS sub-cohort aged 18–60 years in 1981 who were retained for analysis, and all others (deceased, refused, lost to follow-up, unable to participate, or with missing data). Physical Activity Longitudinal Study 1981–2002/04.

	Participants with complete data on LTPA	Participants deceased, refused, lost to follow-up, unable to participate, or with missing data for LTPA
**SELECTED CHARACTERISTICS**	(N = 884)	(N = 1574)
	**n (%)^1^**	**n (%)^1^**
**Male**	390 (44.1)	740 (47.0)
		
1981 Age***		
18–27	288 (32.6)	409 (26.0)
28–39	360 (40.7)	493 (31.3)
40–60	236 (26.7)	672 (42.7)
		
1988 Education***		
Secondary incomplete	143 (16.2)	423 (31.5)
Secondary/Post-secondary	490 (55.6)	681 (50.8)
Completed University	249 (28.2)	238 (17.7)
		
1981 Household income**		
Low (< $15 K)	138 (15.6)	279 (17.7)
Average($15–29 999)	340 (38.5)	501 (31.8)
High (≥ $30 K)	259 (29.3)	337 (21.4)
Missing	147 (16.6)	457 (29.1)
		
Degree of urbanization*		
Large city	420 (47.9)	696 (54.1)
Other urban area	203 (23.2)	269 (20.9)
Rural area	253 (28.9)	322 (25.0)
		
**1981 LTPA**		
Inactive (<1.5 kkd)	483 (54.6)	706 (59.3)
Moderately active (1.5 to <3.0 kkd)	196 (22.2)	224 (18.8)
Active (≥ 3 kkd)	205 (23.2)	260 (21.8)
		
**1981 BMI**		
≤ 25	508 (67.3)	668 (63.1)
> 25	247 (32.7)	390 (36.9)
		
1981 Smoking status***		
Current	318 (36.0)	769 (48.9)
Ex	182 (20.6)	381 (24.2)
Never	384 (43.4)	424 (26.9)
		
1981 Self-rated health***		
Very Good/Excellent	612 (69.3)	941 (62.1)
Good/Fair/Poor	271 (30.7)	575 (37.9)

1. Percentages computed excluding missing data unless otherwise indicated

*: p < 0.05, **: p < 0.01; ***: p < 0.001, for differences based on t-test or chi-square test with (# categories -1) degrees of freedom.

LTPA = Leisure Time Physical Activity; kkd = kilocalories/kilogram/day

### Measures

#### Physical activity indicator

LTPA was assessed in 1981, 1988 and 2002/04 using adaptations of the Minnesota Leisure Time Physical Activity Questionnaire,[11] in which respondents self-reported the frequency and duration of 24 specific activities and up to three volunteered activities over the past year. Activities related to occupation and to household chores are excluded. Average daily energy expenditure (EE), expressed in kcal·kg^-1^·day^-1 ^was computed by summing the products of the metabolic cost of each activity, its average duration, and the number of occasions across the 12-month period, and then dividing by 365, i.e.:

Average daily EE = [∑_*activity *_(METs_*i*_)·(average duration in hours_*i*_)·(total number of occasions over 12 months_*i*_)]/365.

The metabolic costs of activities were developed by an expert panel in 1981 and expressed as multiples of basal resting energy or METs (metabolic equivalents).[12] The measure of EE has criterion validity ranging from ρ = 0.30–0.45, which is typical for physical activity measurement studies, and very good 1-month test-retest reliability (0.91).[13]

Unusually high values of EE were censored at 12 kcal·kg^-1^·day^-1 ^(i.e. values of EE greater than 12 were set to 12); fewer than 2 percent of participants were affected by this transformation at one or more assessment times. The natural logarithm of (EE+1) was used in all regression analyses due to the skewness of the untransformed variable (with one added in order to obtain only non-negative values). For sample description purposes, LTPA was categorized as insufficiently active (EE<1.5 kcal·kg^-1^·day^-1^), moderately active (EE = 1.5-<3 kcal·kg^-1^·day^-1^), and sufficiently active to realize health benefits (EE≥ 3 kcal·kg^-1^·day^-1^), where an EE of 3 kcal·kg^-1^·day^-1 ^is approximately equivalent to the average caloric expenditure of walking one hour per day.

#### Other measures

Baseline age was by categorized by approximate tertile, i.e. 18–27 years, 28–39 years, and 40–60 years. Degree of urbanization was defined according to Statistics Canada definitions  and categorized as large city, other urban, and rural. Education was described using three indicator variables, which were based on the highest level of education reported either in 1981 or in 1988: i) elementary education only or incomplete secondary education; ii) completed secondary education and/or obtained some post-secondary education (in a university, college, or professional trade) without obtaining a university degree; and iii) completed university degree. Baseline (1981) income was based on the question *Approximately what was your family's total income last year, before taxes? ($5,000, $5000–$9,999, $10,000–$14,999, $15,000–$24,999, $25,000–$29,000, $30,000–$35,000, >$35,000, don't know)*. Four indicator variables were created: high (≥ $30 000), average ($15 000–$29 999), low (< $15 000), and missing. Several indicators of health status were included to describe participants, including body mass index (BMI), smoking status, and self-rated health. Height and weight were measured in 1981 according to a standard protocol,[14] and BMI was computed as (weight in kilograms)/(height in meters)^2; ^categories of normal baseline weight (baseline BMI<25), or overweight (BMI> = 25) were created. Participants described their smoking history in 1981, and were categorized as never, ex- or current smokers. Finally, participants were asked to self-rate their general level of health, and were categorized as reporting 'Excellent/Very Good' vs. 'Good/Fair/Poor' health in 1981.

### Analysis

To identify longitudinal trajectories of LTPA beginning in 1981, latent class growth analysis, and more specifically semi-parametric group-based modeling, was used to identify major classes of trajectories (Proc TRAJ in SAS).[15,16] Latent class analysis is recommended when it is suspected that a single set of parameters (such as the mean and slope) would not describe the variables of interest adequately; instead, several underlying (but unknown) 'groups', each with their own set of parameters, are allowed for. Latent class growth analysis is an extension of latent class analysis that can directly model individual trajectories. Latent class growth analysis is a special case of growth mixture modeling with variance and covariance estimates for growth factors set to zero; thus, all growth trajectories within a class are assumed to be identical.[17]. Using the continuous log-transformed measure of LTPA, we allowed for any number of underlying classes, and up to a quadratic polynomial function for each class. Model selection was based on the Bayesian Information Criterion (BIC) as a measure of goodness of fit.[16] After identifying the optimal number of classes, subjects were assigned to the class for which he or she had the highest prior probability. Equations describing the 22 year trajectory for each LTPA class were plotted. For ease of comprehension, illustrated trajectories within each class are based on untransformed data.

To identify independent predictors of trajectory class membership, polytomous logistic regression was used, an extension to logistic regression that permits modeling a nominal dependent variable with more than two categories (in this case, LTPA class). We used the generalized logit function PROC SURVEYLOGISTIC in SAS (Ver. 9.1) to control for clustering within families.

## Results

### Subject characteristics

Table 1 compares selected baseline characteristics of subjects retained for the current analysis (n = 884) and those who were eligible based on their age, and participation in both 1981 and 1988 (n = 1574) but who were excluded for reasons described previously. Participants retained for analysis were younger, had more favorable distributions with respect to education and income, and were less likely to be smokers. There were no differences with respect to baseline physical activity level or overweight status, but those retained for analysis were significantly more likely to view their general level of health favourably (Table 1).

### Latent class analysis

**Figure 1:**
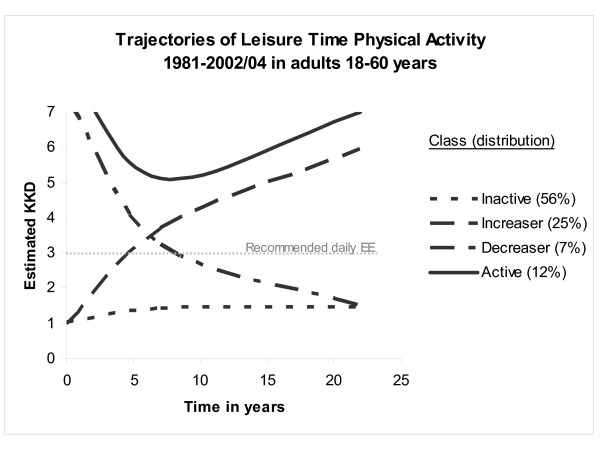
**The four trajectory classes of leisure time physical activity: consistently active, consistently inactive, decreasers, and increasers.** Physical Activity Longitudinal Study 1981–2002/04. (n = 884).

To identify the optimal number of classes, we initially modeled two groups; we then systematically included one additional group, and compared the goodness of fit statistics of each incremental pair (i.e. models with 3 Vs. 2 classes, 4 Vs. 3 classes, 5 Vs. 4 classes, etc). Based on this procedure, the quadratic polynomial growth model with four trajectory classes was retained as the best-fitting model. Median prior probabilities ranged from 0.71 to 0.95 across the four classes. Thirty-seven subjects were assigned to a class for which their highest probability was below 0.50. They exhibited no specific pattern, and other than a slight loss of precision, findings with and without these subjects were almost identical. The four distinct classes are characterized by *consistently inactive*, *consistently active*, *increasing*, and *decreasing *trajectories, and included 56.0, 11.7, 25.2, and 7.1 percent of participants, respectively. Persons in the *consistently inactive *class were characterized by low levels of leisure time physical activity, and little change over time. *Consistently active *participants exhibited trajectories that remained well above recommended levels of 3 kkd (illustrated by the grey dashed line in Figure 1) at all 3 assessments. *Increasers *started below but finished above the recommended threshold, while the reverse was observed for *decreasers*. Estimated growth curves for each class are illustrated in figure 1.

### Predictors of trajectory class membership

We examined five socio-economic and demographic predictors of trajectory class in multivariable analysis, including age, sex, degree of urbanization at baseline, highest reported education, and baseline family income. *Consistently inactive *was selected to be the reference category (Table 2). In addition, we examined the predictors of a *decreasing *versus *consistently active *trajectory. All potential predictors were included in all polytomous logistic regression models.

**Table 2:** Independent predictors of leisure time physical activity trajectory class membership. Physical Activity Longitudinal Study 1981–2002/04.

	**Active Vs. Inactive**	**Decreaser Vs. Inactive**	**Increaser Vs. Inactive**
	AOR (95% CI)	AOR (95% CI)	AOR (95% CI)
**Sex**			
Male (ref)	-	-	-
Female	**0.38 (0.25–0.58)**	**0.37 (0.20–0.66)**	**0.62 (0.44–0.86)**
**Age (years)**			
18–27 (ref)	-	-	-
28–39	**0.44 (0.26–0.74)**	**0.43 (0.22–0.83)**	1.04 (0.69–1.56)
40–60	**0.51 (0.26–0.99)**	**0.35 (0.15–0.81)**	1.48 (0.93–2.35)
**Highest reported education**			
Completed University (ref)	-	-	-
Completed Secondary (<Uni.)	**0.38 (0.24–0.61)**	0.81 (0.44–1.49)	0.94 (0.62–1.42)
Secondary incomplete	**0.39 (0.17–0.87)**	0.85 (0.32–2.22)	1.28 (0.75–2.19)
**1981 Household income**			
High (ref)	**-**	-	-
Average	**0.56 (0.32–0.97)**	0.63 (0.30–1.31)	0.93 (0.61–1.40)
Low	**0.30 (0.13–0.67)**	0.97 (0.44–2.14)	0.55 (0.30–1.01)
Missing	0.59 (0.28–1.25)	1.77 (0.51–2.70	0.74 (0.43–1.27)
**1981 Degree of urbanization**			
Large city (ref)	-	-	-
Other urban area	0.91 (0.48–1.72)	1.18 (0.65–2.14)	0.91 (0.60–1.37)
Rural area	1.08 (0.62–1.89)	**0.41 (0.18–0.95)**	0.77 (0.51–1.15)

Models include all variables in the Table. Estimates significant at p < 0.05 are in bold.

*. Polytomous logistic regression analysis with "Consistently inactive" as the reference class and controlling for clustering within families.

AOR = Adjusted Odds Ratio

#### Predictors of being consistently active Vs. inactive

Women and older participants were less likely than their male and younger counterparts, respectively, to follow a *consistently active *than a *consistently inactive *trajectory. Those with lower educational attainment also experienced lower odds of following a *consistently active *trajectory. A dose-response effect was observed for family income, with those in the mid- and low-income categories having lower odds of being *consistently active *compared with participants in the highest income category, even after controlling for all other individual characteristics (Table 2).

#### Predictors of being a decreaser Vs. consistently inactive

Similar to findings contrasting active and inactive trajectories, women and older participants were less likely than their male and younger counterparts, respectively, to follow a *decreasing *rather than a *consistently inactive *trajectory. Neither education nor income was related to a *decreasing *trajectory. However, the odds of being a *decreaser *were significantly lower among those living in rural areas (Table 2).

The odds of being an *increaser *rather than *consistently inactive *were significantly lower among women compared with men (OR = 0.62). While no other predictors were statistically significant at the 0.05 threshold value, low income participants were much less likely than high income participant to be *increasers *(OR = 0.55; 95 percent confidence interval: 0.30–1.01) (Table 2).

#### Predictors of being a decreaser Vs. consistently active

Participants who were disadvantaged with respect to education and income were more likely to follow a *decreasing *rather than a *consistently active *trajectory, compared with participants who were university educated and those who reported higher family income. In particular, those with low (Vs. high) family income were more than 3 times as likely to be *decreasers *(OR = 3.23, 95% C.I. = 1.18–8.80). In addition, compared with participants who lived in large urban areas in 1981, those who lived in rural areas were significantly less likely to follow a *decreasing *trajectory (OR = 0.38, 95% C.I. = 0.16–0.95). (data not shown).

## Discussion

To our knowledge, this is the first study to identify distinct classes of long term trajectories of LTPA in a diverse sample of adults using latent class growth analysis. Conceptualizing physical activity involvement as a dynamic behavioural process rather than a static risk category, we used growth modeling to identify groups of individuals with vastly differing behavioural trajectories; this allowed us to explore meaningful contrasts and, ultimately, to identify predictors of trajectory class membership. Indeed, observational studies show that tracking of physical activity over the life span is moderate at best, underscoring the need for repeated assessments. [18-23] While cross-sectional designs that incorporate past experience can examine reported change in behaviours, recall of past physical activity involvement is generally poor. [24-26] Even prospective studies have generally employed simple methods to categorize individuals, for example by averaging repeated assessments of physical activity, or through modeling change in physical activity relative to some baseline value.[27] Some prospective studies incorporate absolute values of change (or meaningful categories thereof), typically grouping individuals into only two [6,28-30] or three [31,32] categories. Trajectory analysis improves on these approaches by fully exploiting the longitudinal nature of the data, incorporating both the time and sequence of assessments to produce estimates. Although only three measures of LTPA were obtained in the current study, and fluctuations in yearly LTPA between assessment periods are unknown, each measure of LTPA has a reference period spanning one year, minimizing seasonal and other short term variations. Furthermore, the 22 year follow-up period provides a unique opportunity for examining patterns of activity over a substantial portion of the life span.

It has previously been reported that the PALS cohort is largely representative of the 1981 population, albeit PALS participants were more educated, and less likely to have very low income, to be underweight or to smoke, compared with those who did not participate in the PALS.[8] The cohort retained for the current analysis was younger, healthier, and enjoyed more favorable social circumstances; thus, estimated trajectories describe the experience of a healthier, more advantaged sub-set of the population that was first surveyed in 1981.[8] Nevertheless, even in this healthy sub-sample of the Canadian population, the majority of subjects were consistently inactive. At the last follow-up, 62 percent of adults were below recommended levels of LTPA (i.e. *consistently inactive *and *decreasers*), a proportion similar to findings from the British Birth Cohort, in which 60 percent of adults failed to meet recommended levels of LTPA at age 42 years.[18] Clearly, increasing population levels of physical activity remain an important target for health promotion. In particular, because so few adults reverse behaviours that are acquired earlier in adulthood, programs should aim to help active youth remain active until early adulthood.

The impact of socio-economic factors on physical activity behaviours has been investigated in longitudinal studies,[18,21,33-36] but the extent to which social factors can influence long term trajectories of LTPA remains unclear. In the CARDIA study, higher average attained education was not associated with declines in physical activity, except in white women, while additional years of education during the seven years of follow-up were associated with declines in physical activity only among men.[21] In the British Birth Cohort study, neither social class at birth nor educational attainment predicted change in physical activity over time.[7] The British household panel survey observed that decreases in physical activity over time were predicted by lower grade occupations but not by income.[36] In our study, a decreasing trajectory of LTPA was strongly predicted by both low family income, and lower education. These findings, albeit based on a limited number of studies, suggest that socio-economic disadvantage may be a particularly important risk factor for declines in physical activity among North American populations.

## Limitations

As is to be expected in studies with long follow-up periods, there is a clear selection bias related to survival, health behaviours, and more favourable social circumstances. It is likely that the *distribution *of trajectory classes would have differed in the entire eligible cohort. However, because our analytic cohort includes a diverse population nevertheless, we were able to identify clearly distinct normative long term trajectories, and to identify risk factors for class membership, both of which are unlikely to be influenced by selection bias.

Estimates of EE are based on self reported LTPA and, as with all self-report tools, are subject to misclassification due to over reporting of activity. In addition, we used absolute METs values for computing EE rather than values adjusted for individual characteristics (such as weight, age, sex, and fitness level). While it is possible that the use of relative METs values could impact total EE, the use of absolute METs values is warranted because the focus of the current investigation was to summarize physical activity behaviour independently of individual characteristics, rather than to quantify actual energy costs.

We did not include physical activity related to occupation; lower SES groups are more likely to have employment that require physical exertion, and it is possible that differences between socioeconomic groups would be attenuated if total rather than LTPA had been estimated. Because our focus was on socioeconomic conditions as a precursor to LTPA trajectories, we did not consider fluctuations in social conditions; future studies could compare the relative importance of changes in these conditions and how they relate to health outcomes.

## Conclusion

Our findings provide evidence that longitudinal patterns of LTPA are strongly predicted by socio-economic and demographic factors. On a population scale, these findings suggest that social inequalities persist and may even be amplified over the life span. Although women were less likely than men to increase their LTPA over time, socio-economic factors were not related to such increases, suggesting that health promotion efforts may have been successful across a broad range of socio-economic groups. Nevertheless, women remain an important target for physical activity promotion, as do disadvantaged groups with respect to income and education, all of whom are more likely to experience unfavorable trajectories of LTPA in adulthood. There is a need for continued efforts that target the prevention of disparities related to physical activity before they become established, ideally during childhood and adolescence.

## Abbreviations

AOR: Adjusted Odds Ratio; BMI: Body Mass Index; CFS: Canada Fitness Survey; CSWB: Campbell's Survey of Well-Being; EE: Energy Expenditure; kkd: kilocalories/kilogram/day; LPTA: Leisure Time Physical Activity; METs: Metabolic equivalents; OR: Odds Ratio; PALS: Physical Activity Longitudinal Study; SES: Socio-economic status.

## Competing interests

The authors declare that they have no competing interests.

## Authors' contributions

TB conceptualized and carried out the analysis, and wrote the manuscript; LG, CC and PK conceived of the original study, acquired the data, helped interpret the findings, and provided substantial revisions of earlier drafts.
